# Twelve‐year sarcopenia trajectories in older adults: results from a population‐based study

**DOI:** 10.1002/jcsm.12875

**Published:** 2021-11-30

**Authors:** Caterina Trevisan, Davide Liborio Vetrano, Riccardo Calvani, Anna Picca, Anna‐Karin Welmer

**Affiliations:** ^1^ Aging Research Center, Department of Neurobiology, Care Sciences and Society Karolinska Institutet and Stockholm University Stockholm Sweden; ^2^ Department of Medicine (DIMED), Geriatrics Division University of Padua Padua Italy; ^3^ Department of Medical Sciences University of Ferrara Ferrara Italy; ^4^ Fondazione Policlinico Universitario A. Gemelli IRCCS Rome Italy; ^5^ Stockholm Gerontology Research Center Stockholm Sweden; ^6^ Division of Physiotherapy, Department of Neurobiology, Care Sciences and Society Karolinska Institutet Stockholm Sweden; ^7^ Women's Health and Allied Health Professionals Theme, Medical Unit Medical Psychology Karolinska University Hospital Stockholm Sweden

**Keywords:** Sarcopenia, Transitions, Prospective study, Aged

## Abstract

**Background:**

The dynamic nature of sarcopenia, including possible transitions between its different stages, is currently unknown. We aimed to explore 12 year transitions through sarcopenia stages and identify factors associated with different sarcopenia trajectories in older adults.

**Methods:**

We included 3219 participants (aged ≥60 years, 35.8% men, 96.4% community‐dwelling) from the SNAC‐K study. No sarcopenia (normal muscle strength and mass), probable sarcopenia (low muscle strength and normal muscle mass), and sarcopenia (low muscle strength and mass) were assessed at baseline and up to 12 years. Such conditions were defined based on a modified version of the EWGSOP2 criteria with muscle strength evaluated through handgrip or chair stand tests, and muscle mass from calf circumference. We estimated 1, 5, and 10 year transition probabilities through continuous‐time multistage Markov modelling. Sociodemographic, lifestyle, and medical factors associated with the likelihood of different transitions were evaluated with proportional intensity models, and the associations' strength was expressed as hazard ratio (HR) and 95% confidence interval (CI).

**Results:**

Participants with no sarcopenia had 10‐year probabilities of 17.1% and 5.1% to develop probable sarcopenia and sarcopenia, and a 40.4% chance of not transitioning. Those with probable sarcopenia had similar 5‐year chances of developing sarcopenia (10.3%) and reverting to no sarcopenia (10.7%). Participants with sarcopenia had chances to revert to probable sarcopenia ranging from 8.2% (at 5 years) to 4.7% (at 10 years), and a 70.9% chance of dying after 10 years. Older age (HR = 1.11, 95% CI: 1.07–1.14), male sex (HR = 1.84, 95% CI: 1.16–2.91), current smoking (HR = 1.84, 95% CI: 1.16–2.91), and higher number of chronic diseases (HR = 1.07, 95% CI: 1.00–1.14) were associated with sarcopenia development, while higher levels of physical activity (HR = 1.84, 95% CI: 1.19–2.84) and cognitive function (HR = 1.17, 95% CI: 1.05–1.31 per each 1‐point increase in the Mini‐Mental State Examination) were associated with subsequent higher reversion rates from probable sarcopenia to no sarcopenia (*P* < 0.05 for all). None of the explored characteristics were associated with sarcopenia reversion to healthier stages.

**Conclusions:**

Sarcopenia appears to be a dynamic condition with possible two‐way transitions between different sarcopenia stages, especially the earliest ones. Timely interventions to improve physical and cognitive function and better control individuals' chronic conditions could help counteract sarcopenia progression.

## Introduction

Sarcopenia is a disorder characterized by a progressive and generalized reduction in muscle mass and function, increasing the risk of negative health‐related outcomes, such as injurious falls, hospital admissions, and mortality.^1^, ^2^ The last consensus of the European Working Group on Sarcopenia in Older People (EWGSOP2) in 2018 made two major revisions to the definition of such condition.^1^ First, sarcopenia has now been recognized as a prototypical chronic condition, in which pathogenic processes leading to a loss in muscle mass and function are likely to start at middle ages and not exclusively occur in older adults. Second, in contrast to the previous sarcopenia criteria, priority for the case identification has been given to the assessment of low muscle strength, which identifies individuals with probable sarcopenia. The combination of low muscle strength with low muscle mass or quality ascertains the presence of sarcopenia. The severity of sarcopenia is then determined by assessing possible impairments in physical performance.

These three steps reflect the continuum that well describes sarcopenia development. However, although the progression towards worse sarcopenia stages is the most frequent scenario, a possible reversion from worse to probable sarcopenia or non‐sarcopenia stages may be hypothesized. As already shown for some syndromes, such as frailty,^3^ the development of sarcopenia may be envisioned as a dynamic process, with both possible worsening and improving transitions.

Recently, extensive literature has focused on predictors of sarcopenia, including several sociodemographic, lifestyle, and health‐related aspects (e.g., chronic diseases, hormonal dysfunctions, and inflammatory status).^2^, ^4^ To our knowledge, however, only one population‐based study has longitudinally evaluated the transitions between sarcopenia stages.^5^ In a cohort of 2928 community‐dwelling septuagenarians from The Health, Aging, and Body Composition Study, potentially modifiable factors, such as physical activity and body mass index, were identified as determinants of transitions to sarcopenia. This seminal evidence provided relevant insights about the process towards sarcopenia development and the factors associated with its dynamic nature. This information may be instrumental to determine the most appropriate factors to target by means of tailored interventions and the time windows when the interventions may have greater efficacy.

In the present study, we aimed to explore 12‐year transitions through sarcopenia stages defined according to the EWGSOP2 sarcopenia criteria, while identifying factors associated with such trajectories in older adults.

## Methods

### Study population

We used data from the Swedish National Study on Aging and Care in Kungsholmen (SNAC‐K). This ongoing prospective population‐based study includes older adults aged ≥60 years living in the Kungsholmen area of Stockholm City (Sweden). The selection of the study participants was performed using random stratified sampling, considering the following age cohorts: 60, 66, 72, 78, 81, 84, 87, 90, 93, 96, and 99+ years (further details can be found in previous publications).^6^, ^7^ The baseline assessment was performed in 2001–2004 and had a participation rate of 73.3%. In order to better capture the occurrence of age‐related changes in multiple health domains, participants in the oldest age cohorts (age ≥78 years) were assessed every 3 years and the youngest ones (age 60–72 years) every 6 years. For this work, of the 3363 participants initially included in the SNAC‐K, we excluded 144 individuals who had incomplete data on muscle mass and/or strength, obtaining a final sample of 3219 participants. Data from baseline to the 12‐year follow‐up were considered.

The SNAC‐K study complies with the principles of the Declaration of Helsinki and was approved by the Regional Ethical Review Board in Stockholm. Written informed consent to participate in the study was collected from all participants or the next of kin for those with cognitive impairment.

### Data collection

Baseline and follow‐up assessments of the study participants were performed by nurses and physicians at the research centre or, in the case of inability to come to the centre, at home. Evaluations included face‐to‐face interviews, reviews of the medical records, physical examinations, and administrations of scales and questionnaires.

#### Sarcopenia

The presence of sarcopenia at baseline and each follow‐up was assessed based on a modified version of the European Working Group's revised criteria on Sarcopenia in Older People (EWGSOP2).^1^ As recommended, we considered muscle strength, muscle mass, and physical performance.

Muscle strength was evaluated by testing handgrip in both hands, and the best result was considered for the analyses. For those participants who had missing data on handgrip, we considered chair stand test results. Low muscle strength was defined as handgrip <27 kg for men and <16 kg for women, or as >15 s for five rises from a chair.^1^ Cohen's *κ* value for the agreement between handgrip and chair stand test in detecting individuals with low muscle strength in our sample was 0.307 (*P* < 0.001).

Low muscle mass was considered as having a calf circumference less than the 20th sex‐specific percentile of our sample,^5^, ^8^ that is, <34 cm for men and <32 cm for women. These values are in line with the cut‐offs for moderately/severely low calf circumference suggested by a recent study.^9^

A walking speed of ≤0.8 m/s was used to define low physical performance. This measure was assessed over 6 m or, for individuals who defined themselves as slow‐walkers or those evaluated at home, 2.4 m. Previous studies demonstrated that evaluations of this parameter over a distance of 6 and 2.4 m in older adults are comparable.^10^

Following the EWGSOP2 algorithm,^1^ we defined ‘no sarcopenia’ as the presence of normal muscle strength and mass, ‘probable sarcopenia’ as the presence of low muscle strength and normal muscle mass, ‘non‐severe sarcopenia’ as the presence of low muscle strength and mass with normal physical performance, and ‘severe sarcopenia’ as the combination of low muscle strength, low muscle mass, and low physical performance. In this study, because of the few participants with non‐severe sarcopenia, we merged the non‐severe and severe sarcopenia conditions into a unique ‘sarcopenia’ category.

#### Mortality

Information on the death dates for those who died over the 12‐year study period was derived from the Swedish Cause of Death Registry.

#### Covariates

The following sociodemographic data were collected from each participant: age, sex, educational level (classified as high school degree or above vs. middle school or below), and living arrangement (classified as living in a nursing home vs. living in the community with a cohabitant vs. living in the community, alone). As regards risk behaviours and lifestyle characteristics, we considered smoking habits (categorized as never smoked vs. former smoker vs. current smoker), alcohol consumption [classified as none or occasional drinker vs. light to moderate (1–14 drinks/week for men and 1–7 drinks/week for women) vs. heavy (≥15/≥8 drinks/week for men and women, respectively)], and physical activity level. For the latter variable, we recorded information on the frequency and intensity of engagement in physical activities. Based on international recommendations,^11^, ^12^ we classified participants as physically inactive if they were engaged in light and/or moderate‐to‐intense activities ≤2–3 times/month, and as physically active if they reported at least a weekly frequency of light or moderate‐to‐intense activities.^13^ As a proxy of nutritional status,^14^ we computed the body mass index (BMI) at baseline from the ratio of participants' body weight and height squared (kg/m^2^). At each assessment, we evaluated cognitive performance through the Swedish version of the Mini‐Mental State Examination (MMSE)^15^ and the number of chronic diseases. Physicians ascertained chronic diseases based on physical examinations, biochemical analyses, reviews of ongoing treatments, and information obtained from national inpatient and outpatient registers.^16^ For this study, we considered either the total number of chronic diseases or the presence of the following disease categories: cardiovascular diseases, neuropsychiatric diseases, gastrointestinal or kidney diseases, respiratory diseases, musculoskeletal diseases, endocrine or hematologic diseases, and cancer (details can be found in Supporting Information, *Appendix* S1).

### Statistical analysis

Baseline characteristics of the sample are reported as mean and standard deviation (SD) or count (%). Characteristics were compared across individuals with no sarcopenia, probable sarcopenia, and sarcopenia through Student's *t*‐test and *χ* ^2^ or Fisher's exact test.

The transition occurrence across different sarcopenia stages (no sarcopenia, probable sarcopenia, and sarcopenia), death, and loss to follow‐up was reported first in absolute values and displayed by mean of an alluvial plot. Second, 1‐, 5‐, and 10‐year transition probabilities and the mean permanence time in each status were estimated through continuous‐time multistage Markov modelling. This analysis considered all the transitions observed for each individual during the 12‐year follow‐up because individuals could have experienced more than one transition. Death and loss to follow‐up were considered as absorbing states. We chose standard time points at 1, 5, and 10 years to derive estimates that could have a higher clinically meaningful prognostic value and may increase comparability with previous studies.^10^, ^11^ Third, we investigated factors associated with individuals' transitions over the observation period through proportional intensity models, and the strength of such associations was expressed as hazard ratios with 95% confidence intervals (for details, please see Jackson and Jackson^17^). Proportional intensity models generalize Cox regressions and allow to perform analyses on recurrent events.^18^ To improve the model convergence in this analysis, we used a quasi‐Newton optimization algorithm (the Broyden–Fletcher–Goldfarb–Shanno) and a discrete‐time model. For this analysis, we selected factors that presented a scientific rationale to support a potential association with sarcopenia transitions, including sociodemographic characteristics (age, sex, and education), risk behaviours (smoking and drinking habits, and physical activity level), cognitive performance (MMSE), nutritional status (baseline BMI), and multimorbidity (number of chronic diseases). These factors were firstly tested separately in unadjusted analyses and, secondly, included simultaneously in the model. For time‐varying variables (physical activity level, MMSE, and the number of chronic diseases), the model considers the value at the beginning of each observed transition. The model provides estimates for all possible transitions from no sarcopenia, probable sarcopenia, and sarcopenia, with participants stable in each of these stages being the reference groups.

As sensitivity analyses, we evaluated the association between categories of chronic diseases and sarcopenia transition probabilities, and after including only data from the 6‐ and 12‐year assessments of the participants. A further sensitivity analysis was performed to investigate factors associated with each transition considering individuals living in the community at baseline.

Analyses were performed using R *alluvial* and *msm* packages.^17^, ^19^, ^20^ All tests were two tailed, and we set a *P*‐value <0.05 for statistical significance.

## Results

The study included 3219 individuals, 35.8% men, with a mean age of 74.2 (SD 11) years. A total of 63.2% among the study participants were non‐sarcopenic at baseline, while 27% had probable sarcopenia, and 9.7% had sarcopenia (1.9% non‐severe and 7.8% severe, data not shown). Of note, the prevalence of sarcopenia was 8.2% among community‐dwelling and 51.3% among institutionalized individuals. *Table* 1 reports the participants' characteristics in the total sample and by sarcopenia status. People with no sarcopenia were more likely to be men, younger, more educated, physically active, and generally healthier than those in the other sarcopenia categories. They were also more likely to be former or current smokers and to have light‐to‐moderate or heavy alcohol consumption compared with the other sarcopenia groups. Focusing on physical performance, the prevalence of low walking speed was 10.1% among those with no sarcopenia, 54.9% among those with probable sarcopenia, and 80.3% among sarcopenic participants (for the prevalence of sarcopenia criteria in male and female participants, please see *Table* S1).

**Table 1:** Characteristics of the sample as a whole and by sarcopenia at baseline

	All	No sarcopenia	Probable sarcopenia	Sarcopenia	*P*‐value
*n* (%)	3219 (100)	2036 (63.2)	869 (27.0)	314 (9.8)	
Sex (male, %)	1153 (35.8)	866 (42.5)	197 (22.7)	90 (28.7)	<0.001
Age (years), mean (SD)	74.20 (10.96)	69.34 (8.52)	80.85 (9.53)	87.50 (7.60)	<0.001
Living arrangement (%)					<0.001
Nursing home	115 (3.6)	8 (0.4)	48 (5.5)	59 (18.8)	
Community, living with cohabitants	1721 (53.5)	954 (46.9)	568 (65.4)	199 (63.4)	
Community, living alone	1383 (43.0)	1074 (52.8)	253 (29.1)	56 (17.8)	
Education: high school or above (%)^a^	2659 (83.1)	1797 (88.3)	642 (74.9)	220 (71.4)	<0.001
Body mass index (kg/m^2^)					<0.001
<18.5	88 (2.7)	22 (1.1)	17 (2.0)	49 (15.6)	
18.5–24.9	1493 (46.4)	860 (42.2)	410 (47.2)	223 (71.0)	
25–29.9	1252 (38.9)	880 (43.2)	332 (38.2)	40 (12.7)	
≥30	386 (12.0)	274 (13.5)	110 (12.7)	2 (0.6)	
Active physical level (%)	2184 (67.8)	1634 (80.3)	454 (52.2)	96 (30.6)	<0.001
Smoking habit (%)^a^					<0.001
Never	1493 (46.4)	857 (42.1)	471 (54.2)	165 (52.5)	
Former	1213 (37.7)	829 (40.7)	290 (33.4)	94 (29.9)	
Current	452 (14.0)	340 (16.7)	83 (9.6)	29 (9.2)	
Alcohol consumption (%)^a^					<0.001
No or occasional	1150 (35.7)	512 (25.1)	429 (49.4)	209 (66.6)	
Light to moderate	1721 (53.5)	1261 (61.9)	386 (44.4)	74 (23.6)	
Heavy	296 (9.2)	259 (12.7)	28 (3.2)	9 (2.9)	
No. of chronic diseases, mean (SD)	3.98 (2.44)	3.26 (2.01)	5.03 (2.53)	5.72 (2.83)	<0.001
MMSE, mean (SD)	27.77 (4.28)	28.99 (1.58)	26.59 (5.19)	23.15 (7.89)	<0.001
Low muscle strength (%)	1183 (36.8)	0 (0.0)	869 (100.0)	314 (100.0)	<0.001
Handgrip (*N*), mean (SD)^a^	256.2 (113.6)	292.6 (102.6)	140.6 (55.3)	147.11 (57.41)	<0.001
Chair stand test (s)^a^	28.5 (27.0)	16.8 (16.8)	44.4 (29.5)	59.90 (25.47)	<0.001
Low muscle mass (%)	458 (14.3)	144 (7.1)	0 (0.0)	314 (100.0)	<0.001
Calf circumference (cm)	35.8 (3.8)	36.8 (3.3)	35.9 (2.9)	29.55 (2.44)	<0.001
Low walking speed (%)	927 (29.0)	203 (10.1)	472 (54.9)	252 (80.3)	<0.001
Walking speed (m/s)	0.97 (0.47)	1.18 (0.33)	0.68 (0.45)	0.39 (0.38)	<0.001

MMSE, Mini‐Mental State Examination; SD, standard deviation.

*P*‐values refer to the comparison between individuals with different sarcopenia status.

^a^ *n* = 18 participants had missing data on education, *n* = 61 on smoking habit, *n* = 52 on drinking habit, *n* = 624 on handgrip, and *n* = 6 on chair stand test.

**Figure 1:**
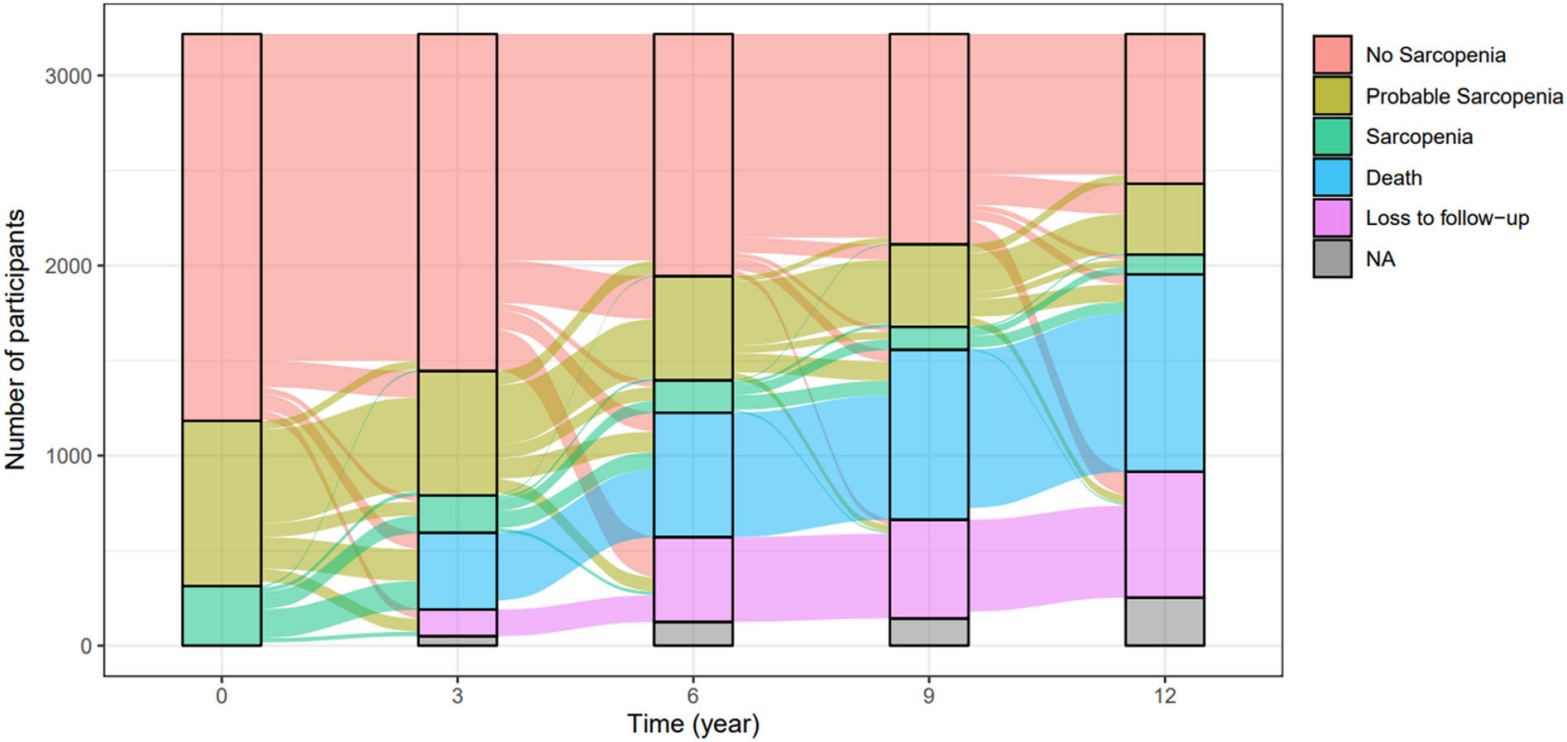
Alluvial plot illustrating the transitions between sarcopenia stages in the SNAC‐K study participants over a 12 year follow‐up (*n* = 3219). NA, not available data on sarcopenia.

*Figure* 1 illustrates the study participants' transitions across sarcopenia stages over the 12‐year follow‐up (the total number of individuals involved in each transition is reported in *Table* S2). Based on these observations, we estimated 1‐, 5‐, and 10‐year transition probabilities. As shown in *Table* 2, the probability of non‐sarcopenic individuals maintaining their status was 90.4% at 1 year and decreased up to 40.4% at 10 years. Conversely, they have 10 year probabilities of 17.1% and 5.1% of developing probable sarcopenia and sarcopenia, respectively. Considering participants with probable sarcopenia, the chance of maintaining their status decreased from 79.3% at 1 year to 14.5% at 10 years. Conversely, their probability of developing sarcopenia reached 10.3% at 5 years, likewise reverting to no sarcopenia (10.7%). The chance of sarcopenic participants to remain sarcopenic ranged from 67.8% to 3.4% at 1 and 10 years, respectively, while the corresponding probabilities of dying increased from 21.4% to 70.9%. The probability of reverting from sarcopenia to probable sarcopenia was 8.2% and 4.7% at 5 and 10 years, respectively, while the 10‐year probability to revert to no sarcopenia was 3.5%.

**Table 2:** Estimated 1‐, 5‐, and 10‐year sarcopenia transition probabilities

	From		
	No sarcopenia	Probable sarcopenia	Sarcopenia
1‐ year probability of transition (%) to			
No sarcopenia	90.4	3.9	1.1
Probable sarcopenia	6.5	79.3	5.2
Sarcopenia	1.0	6.7	67.8
Death	0.9	5.7	21.4
Loss to follow‐up	1.3	4.4	4.6
5‐ year probability of transition (%) to			
No sarcopenia	62.0	10.7	3.4
Probable sarcopenia	17.4	34.3	8.2
Sarcopenia	4.2	10.3	15.6
Death	8.4	27.7	59.1
Loss to follow‐up	8.0	16.9	13.7
10‐year probability of transition (%) to			
No sarcopenia	40.4	10.7	3.5
Probable sarcopenia	17.1	14.5	4.7
Sarcopenia	5.1	5.6	3.4
Death	21.0	44.2	70.9
Loss to follow‐up	16.4	25.0	17.5

Probabilities are estimated considering all observed transitions over the 12 year follow‐up, using continuous‐time multistate Markov models.

Overall, the mean permanence time in each status before experiencing any transition was 9.69 years for no sarcopenia, 4.22 years for probable sarcopenia, and 2.55 years for sarcopenia (*Table* S3).

*Table* 3 shows the factors associated with sarcopenia transitions in our sample (for the univariate analyses and for transitions to loss to follow‐up, please see *Tables* S4 and S5). Factors directly associated with the progression of no sarcopenia to worse sarcopenia stages were older age, male sex, current smoking, and a higher number of chronic diseases. Considering BMI, each 1 kg/m^2^ increase was associated with a higher probability of progressing from no sarcopenia to probable sarcopenia and a lower chance of developing sarcopenia. For probable sarcopenia, older age and male sex were positively associated with the likelihood of developing sarcopenia, while inverse associations were observed for former smoking habits and higher BMI. The chance of reverting from probable to no sarcopenia decreased with older age and a higher number of chronic diseases. At the same time, it increased with higher physical activity and cognitive function. For sarcopenic individuals, no factors were significantly associated with the chance of reverting to probable or no sarcopenia. In all sarcopenia groups, older age, male sex, and a higher number of chronic diseases were associated with higher mortality, while the contrary was observed for an active physical level and higher cognitive function. No substantial differences were observed after excluding institutionalized individuals (*Table* S6), as well as after including only the 6‐ and 12‐year assessments of the participants (data not shown). Among the categories of chronic conditions, the presence of musculoskeletal diseases was associated with a higher risk of developing probable sarcopenia, while cancer seemed to increase both the probability of progressing to and reversing from probable sarcopenia (*Table* S7).

**Table 3:** Factors associated with transitions from no sarcopenia, probable sarcopenia, and sarcopenia

	Multivariable hazard ratio (95% confidence interval) of transition								
	From no sarcopenia to			From probable sarcopenia to			From sarcopenia to		
	Probable sarcopenia	Sarcopenia	Death	No sarcopenia	Sarcopenia	Death	No sarcopenia	Probable sarcopenia	Death
Age (years)	**1.08 (1.07–1.10)**	**1.11 (1.07–1.14)**	**1.07 (1.05–1.08)**	**0.98 (0.96–1.00)**	**1.04 (1.01–1.06)**	**1.07 (1.05–1.08)**	0.99 (0.93–1.06)	0.96 (0.93–1.00)	**1.04 (1.02–1.07)**
Sex (male vs. female)	**0.73 (0.60–0.90)**	**1.84 (1.16–2.91)**	**1.83 (1.42–2.34)**	0.93 (0.61–1.40)	**1.99 (1.31–3.01)**	**1.49 (1.16–1.91)**	0.77 (0.22–2.65)	0.48 (0.19–1.19)	**2.00 (1.45–2.77)**
Education (high vs. low)	1.30 (0.98–1.72)	1.07 (0.59–1.92)	1.15 (0.83–1.60)	0.87 (0.55–1.37)	1.04 (0.64–1.68)	0.80 (0.63–1.02)	1.21 (0.33–4.42)	1.42 (0.58–3.47)	0.87 (0.63–1.19)
Smoking habits (ref: never)									
Former	0.86 (0.71–1.04)	1.32 (0.81–2.16)	1.11 (0.85–1.44)	0.84 (0.60–1.20)	**0.65 (0.43–0.98)**	1.10 (0.88–1.37)	0.55 (0.14–2.07)	0.58 (0.26–1.31)	1.03 (0.75–1.42)
Current	0.89 (0.67–1.19)	**2.13 (1.22–3.74)**	**1.98 (1.45–2.71)**	0.96 (0.56–1.62)	0.77 (0.38–1.57)	**1.76 (1.22–2.52)**	1.15 (0.29–4.59)	0.51 (0.15–1.74)	1.10 (0.69–1.74)
Alcohol (ref: no/occasional)									
Light to moderate	1.06 (0.87–1.31)	0.93 (0.57–1.51)	**0.75 (0.57–0.97)**	1.44 (0.99–2.10)	1.05 (0.71–1.56)	0.84 (0.67–1.04)	0.81 (0.28–2.35)	1.75 (0.88–3.47)	0.77 (0.57–1.05)
Heavy	0.88 (0.59–1.32)	0.85 (0.35–2.05)	0.82 (0.55–1.22)	0.77 (0.28–2.11)	0.75 (0.28–2.01)	1.06 (0.67–1.68)	0.93 (0.08–10.25)	1.77 (0.31–9.97)	0.60 (0.29–1.25)
Physical level (active vs. inactive)	0.97 (0.77–1.21)	0.73 (0.44–1.20)	**0.52 (0.41–0.67)**	**1.84 (1.19–2.84)**	0.82 (0.56–1.21)	**0.77 (0.62–0.95)**	0.86 (0.29–2.53)	2.01 (0.92–4.41)	**0.74 (0.55–1.00)**
MMSE	1.03 (0.97–1.08)	0.96 (0.87–1.06)	**0.91 (0.87–0.95)**	**1.17 (1.05–1.31)**	1.03 (0.96–1.11)	**0.93 (0.90–0.95)**	1.17 (0.92–1.48)	1.01 (0.91–1.12)	**0.95 (0.92–0.97)**
BMI (kg/m^2^)	**1.05 (1.02–1.07)**	**0.75 (0.70–0.80)**	0.98 (0.95–1.01)	1.00 (0.96–1.04)	**0.86 (0.81–0.91)**	**0.98 (0.95–1.00)**	1.07 (0.90–1.27)	1.10 (0.98–1.22)	**0.94 (0.89–0.99)**
No. of chronic diseases	1.03 (0.99–1.06)	**1.07 (1.00–1.14)**	**1.07 (1.03–1.11)**	**0.95 (0.89–1.00)**	1.04 (0.98–1.09)	**1.06 (1.03–1.09)**	0.89 (0.76–1.05)	1.03 (0.95–1.12)	**1.08 (1.04–1.12)**

BMI, body mass index; MMSE, Mini‐Mental State Examination.

High educational level includes high school degree or above. Physical level, Mini‐Mental State Examination, and the number of chronic diseases are included in the model as time‐varying variables. Participants stable in no sarcopenia, probable sarcopenia, and sarcopenia stages are the reference categories. Bold values indicate statistically significant estimates (*P*‐value <0.05). For transitions to loss to follow‐up, please see *Table* S5.

## Discussion

The present study supports a view of sarcopenia as a dynamic condition with both progressions and reversions across different sarcopenia stages. Modifiable and non‐modifiable factors were associated with the chance of experiencing transitions towards progression or reversion starting from no sarcopenia and probable sarcopenia stages. Conversely, none of the factors here analysed seemed to influence the chance of reverting from sarcopenia to less severe stages.

The prevalence of sarcopenia in our sample was in line with that reported by previous studies in similar populations and settings, in both Western and Eastern countries.^21^, ^25^ However, some other works reported slightly lower estimates than ours,^26^, ^27^ probably because of the different methods and cut‐off values used to assess low muscle mass and strength. In our study, we assessed participants' calf circumference, as a proxy estimate of muscle mass,^1^ which could make the comparison of sarcopenia derived from other more accurate parameters difficult.

After evaluating the changes across sarcopenia stages experienced by the study participants over 12 years, we estimated short‐term, medium‐term, and long‐term transition probabilities. Interestingly, at 1 year, most individuals tended to remain in their status, especially those with no or probable sarcopenia. The picture changed in the longer term; that is, the 10‐year chances for non‐sarcopenic and probable sarcopenic individuals of staying stable in their stages decreased up to 40.4% and 14.5%, respectively. Although the most frequent trend was the progressive development of the condition, probable sarcopenia seemed to be the most dynamic stage, with a 10% chance both of progressing to sarcopenia and of reversing to no sarcopenia at 5 years. Conversely, sarcopenia showed lower 5‐ and 10‐year probabilities of remaining stable but a more marked likelihood of dying compared with less severe states.

Murphy *et al*.^5^ showed that people aged 70–79 years tended to persist in their initial sarcopenia status over 9 years. Moreover, the probabilities of transitions towards worse sarcopenia stages ranged from 3% to 7%, and the reversions from pre‐sarcopenia/sarcopenia to more normal states were even less frequent. The discrepancies with our results could be due to differences in the study population, which was not restricted to well‐functioning septuagenarians (indeed, in our study, older age was consistently associated with sarcopenia progression), the length of follow‐up, and the definition of the various sarcopenia stages.^5^

When looking at the factors associated with sarcopenia transitions, we found that older age, male sex, current smoking habits, and a higher number of chronic diseases were associated with a greater risk of sarcopenia progression. Among the different types of chronic conditions, musculoskeletal diseases and cancer seemed to increase the chance of developing probable sarcopenia. Surprisingly, cancer was also associated with a higher probability of reversing from probable sarcopenia to normal stages. The latter result, however, could be affected by the scarce number of individuals with cancer in the study and by selective survival bias. Overall, our findings corroborate previous works focusing on factors that might accelerate damage in muscle mass and function. Mechanisms behind such detrimental effects include prolonged states of chronic inflammation, oxidative stress, and mitochondrial alterations.^2^, ^4^, ^28^ These aspects contribute to the establishment of unbalances between protein anabolism and catabolism^2^, ^4^ and could also affect the neuromuscular junction integrity, along with age‐related neurodegenerative processes.^28^, ^29^

In addition to advanced age and male sex,^5^, ^30^, ^31^ smoking has also been identified as a sarcopenia risk factor,^30^, ^33^ although with some inconsistencies.^32^, ^34^ In our study, current smoking was associated with a higher chance of progressing from no sarcopenia to sarcopenia, while we observed the opposite effect for former smoking habits in individuals with probable sarcopenia. However, the latter finding could be linked to the fact that those who quitted smoking over their life course may have also changed other unhealthy behaviours (e.g. sedentarism), eventually preventing sarcopenia development. Contrasting results also emerged for BMI, which is included among the phenotypic criteria for malnutrition according to current guidelines.^14^ Indeed, we observed that each 1‐ unit increase in baseline BMI was associated with a 5% higher probability of progressing from no sarcopenia to probable sarcopenia, but with a 25% lower chance of developing sarcopenia. These opposite effects are likely to be influenced by the initial BMI of non‐sarcopenic and probable sarcopenic individuals in our sample. Indeed, the former had more frequent excess weight conditions that may alter muscle quality and function.^35^ Furthermore, the negative effect of underweight and malnutrition on muscle health and sarcopenia risk has been pointed out in several studies.^31^, ^33^, ^34^

Concerning chronic diseases, we found that individuals with a higher number of chronic conditions were more likely to progress to sarcopenia and had a lower chance of reverting from probable sarcopenia to normal status. Moreover, people who were more physically active and cognitively intact also had a higher probability of getting back from probable sarcopenia to normal status. In line with these findings, previous reports observed that sarcopenia was associated with long‐term conditions and multimorbidity,^36^, ^37^ as well as low physical activity levels^2^, ^4^, ^30^, ^31^, ^38^ and lower cognitive performance.^39^ However, the novelty of our results lies in the impact that these factors may have not only on sarcopenia progression but also on the reversion from its initial stages. This suggests that intervention aimed at preventing and/or treating chronic diseases and preserving high physical activity and cognitive performance may help counteract sarcopenia and reverse its course, especially at the earlier stages. As emerged from our results, although several factors seemed to influence the chance of reversion from probable to no sarcopenia, this was not the case for sarcopenia, whose reversion to better stages was less frequent and, more importantly, not associated with any factor considered. Indeed, a recent study evaluated the 1 year trends of sarcopenia after hip fracture and showed no reduction in its prevalence over time, reinforcing the need for interventions contrasting sarcopenia before the occurrence of its more detrimental consequences (e.g. falls and fractures).^40^

Limitations of our study include the use of EWGSOP2 criteria modified for the assessment of low muscle mass, which was derived from the calf circumference measure. While it is widely acknowledged that anthropometric measures are less accurate than other body composition techniques, such as bioelectric impedance analysis and dual‐energy X‐ray absorptiometry, calf circumference may still represent a good proxy for muscle mass estimation in contexts where no other diagnostic methods are available.^1^, ^9^ Moreover, we could not consider separately non‐severe and severe sarcopenia conditions due to the few individuals having non‐severe sarcopenia. Similarly, our study population was composed of less than 4% institutionalized individuals; therefore, we could not provide robust estimates for those living in nursing homes. Finally, we did not consider dietary and nutritional intakes among the factors associated with sarcopenia transitions, but this issue will be subject of future investigations. On the other hand, strengths of this work include the large study population and the long follow‐up with frequent assessments at 3‐ or 6‐year intervals. Moreover, the use of a standard definition of sarcopenia and of advanced statistical analyses to estimate the probabilities of transitions across different stages of the disease reinforce the findings and novelty of this study.

In conclusion, sarcopenia is a dynamic condition that seems to progress slowly in its initial stages, with possible reversions to normal status. Higher levels of physical activity and cognitive performance may be associated with a higher chance of reverting from probable sarcopenia to no sarcopenia, while multimorbidity seems to be associated with transitions towards sarcopenia development. This supports the need for implementing early interventions to preserve physical and cognitive function and manage individuals' chronic conditions. Future investigations are needed to uncover interventions effective also in individuals with overt sarcopenia.

## Conflict of interest

None declared.

## Funding

The SNAC‐K study was supported by the Ministry of Health and Social Affairs, Sweden (Socialdepartementet); the participating county councils and municipalities; and the Swedish Research Council [Vetenskapsrådet (VR)]. A.‐K.W. was supported by specific grants from VR (Grant Number 521‐2014‐21‐96) and the Swedish Research Council for Health, Working Life and Welfare [Forskningsrådet om Hälsa, Arbetsliv och Välfärd (FORTE), Grant Number 2018‐01888].

## Supporting information


**Appendix S1.** List of chronic diseases considered.
**Table S1.** Criteria for sarcopenia definition in male and female participants.
**Table S2.** Total number of observed transitions in participants categorized by sarcopenia over a 12‐year follow‐up.
**Table S3.** Mean permanence time in each sarcopenia state.
**Table S4.** Factors associated with transitions from no sarcopenia, probable sarcopenia, and sarcopenia (univariate analysis).
**Table S5.** Factors associated with transitions from no sarcopenia, probable sarcopenia, and sarcopenia to loss to follow‐up.
**Table S6.** Factors associated with transitions from no sarcopenia, probable sarcopenia, and sarcopenia in community‐dwelling individuals (*n* = 3,104).
**Table S7.** Chronic diseases associated with transitions from no sarcopenia, probable sarcopenia, and sarcopenia.
