# Supplementary material for: Twelve‐year sarcopenia trajectories in older adults: results from a population‐based study
Source: J Cachexia Sarcopenia Muscle. 2021 Nov 30;13(1):254–63. doi: 10.1002/jcsm.12875 (PMC8818646; doi:10.1002/jcsm.12875)
Supplement: Supplementary file 1 — Appendix S1. List of chronic diseases considered. Table S1. Criteria for sarcopenia definition in male and female participants. Table S2. Total number of observed transitions in participants categorized by sarcopenia over a 12‐year follow‐up. Table S3. Mean permanence time in each sarcopenia state. Table S4. Factors associated with transitions from no sarcopenia, probable sarcopenia, and sarcopenia (univariate analysis). Table S5. Factors associated with transitions from no sarcopenia, probable sarcopenia, and sarcopenia to loss to follow‐up. Table S6. Factors associated with transitions from no sarcopenia, probable sarcopenia, and sarcopenia in community‐dwelling individuals (n = 3,104). Table S7. Chronic diseases associated with transitions from no sarcopenia, probable sarcopenia, and sarcopenia. [file JCSM-13-254-s001.docx]

**S1 Appendix. List of chronic diseases considered**

The following chronic diseases were assessed by physicians from physical examination, blood tests, review of medicines used, and national health registers[1]: anemia; venous and lymphatic diseases; blood and blood forming organ diseases; autoimmune diseases; thyroid diseases; hypertension; ischemic heart disease; heart failure; peripheral vascular disease; atrial fibrillation; cardiac valve diseases; bradycardias and conduction diseases, other cardiovascular diseases; chronic infectious diseases, chronic kidney diseases; other genitourinary diseases; chronic pancreas, biliary tract and gallbladder diseases; colitis and related diseases; allergy; asthma; COPD, emphysema, chronic bronchitis; other respiratory diseases; diabetes; esophagus, stomach and duodenum diseases; colitis and related diseases; inflammatory bowel diseases; other digestive diseases; hypercholesterolemia; obesity; chronic liver diseases; other metabolic diseases; blindness, visual impairment; cataract and other lens diseases; glaucoma; other eye diseases; deafness, hearing impairment; ear, nose, throat diseases; chronic ulcer of the skin; other skin diseases; inflammatory arthropathies; osteoarthritis and other degenerative joint diseases; osteoporosis; dorsopathies; migraine and facial pain syndromes; neurotic, stress-related and somatoform diseases; cerebrovascular disease; other neurological diseases; multiple sclerosis; peripheral neuropathy; epilepsy; sleep disorders; depression and mood diseases; other psychiatric and behavioral diseases; schizophrenia and delusional diseases; Parkinson and parkinsonism; prostate diseases; solid neoplasms; hematological neoplasms; and, chromosomal abnormalities.

For the sensitivity analysis on the association between chronic diseases and sarcopenia trajectories, we considered the following categories:

- Cardiovascular diseases, e.g. atrial fibrillation, bradycardias or other conduction disorders, cardiac valve diseases, heart failure, ischemic heart diseases, peripheral vascular diseases.

- Neuropsychiatric diseases, e.g. cerebrovascular diseases, epilepsy, multiple sclerosis, peripheral neuropathy, Parkinson's disease or parkinsonism, dementia, schizophrenia and delusional disorders, behavioral disorders, depression or mood disorders, neurotic stress-related and somatoform disorders.

- Gastrointestinal/kidney diseases, e.g. chronic kidney disease, disorders in the esophagus, stomach or duodenum, chronic pancreatitis, chronic biliary disorders, inflammatory bowel diseases, colitis-related or other digestive disorders, chronic liver disease.

- Respiratory diseases, e.g. allergy, asthma, COPD, and other respiratory disorders.

- Musculoskeletal diseases, e.g. dorsopathies, osteoarthritis and degenerative joint diseases, osteoporosis, inflammatory arthropathies, and other musculoskeletal and joint diseases (excluding sarcopenia).

- Endocrine/hematologic diseases, e.g. thyroid diseases, diabetes, anemia, venous or lymphatic diseases, and blood diseases.

**REFERENCES**

[1] A. Calderón-Larrañaga, D.L. Vetrano, G. Onder, L.A. Gimeno-Feliu, C. Coscollar-Santaliestra, A. Carfí, M.S. Pisciotta, S. Angleman, R.J.F. Melis, G. Santoni, F. Mangialasche, D. Rizzuto, A.-K. Welmer, R. Bernabei, A. Prados-Torres, A. Marengoni, L. Fratiglioni, Assessing and Measuring Chronic Multimorbidity in the Older Population: A Proposal for Its Operationalization, Journals Gerontol. Ser. A Biol. Sci. Med. Sci. (2016) glw233. https://doi.org/10.1093/gerona/glw233.

**S1 Table. Criteria for sarcopenia definition in male and female participants**

|  | **No**  **Sarcopenia** | **Probable Sarcopenia** | **Sarcopenia** | **p-value** |
| --- | --- | --- | --- | --- |
| **Male participants (n=1153)** |  |  |  |  |
| n (%) | 866 (75.1) | 197 (17.1) | 90 (7.8) |  |
| Low muscle strength (%) | 0 (0.0) | 197 (100.0) | 90 (100.0) | <0.001 |
| Handgrip (N, mean [SD]) | 388.0 (77.2) | 215.8 (42.8) | 202.0 (42.8) | <0.001 |
| Chair stand test (sec) | 15.0 (14.7) | 43.4 (29.9) | 51.70 (29.0) | <0.001 |
| Low muscle mass (%) | 69 (8.0) | 0 (0.0) | 90 (100.0) | <0.001 |
| Calf circumference (cm) | 37.5 (3.0) | 37.0 (2.6) | 30.9 (2.1) | <0.001 |
| Low walking speed (%) | 65 (7.5) | 87 (45.1) | 59 (65.6) | <0.001 |
| Walking speed (m/s) | 1.23 (0.32) | 0.74 (0.41) | 0.53 (0.41) | <0.001 |
|  |  |  |  |  |
| **Female participants (n=2066)** |  |  |  |  |
| n (%) | 1170 (55.6) | 672 (33.7) | 224 (10.8) |  |
| Low muscle strength (%) | 0 (0.0) | 672 (100.0) | 224 (100.0) | <0.001 |
| Handgrip (N, mean [SD]) | 220.3 (43.3) | 116.2 (32.1) | 112.25 (33.00) | <0.001 |
| Chair stand test (sec) | 18.1 (18.1) | 44.7 (29.4) | 63.20 (23.15) | <0.001 |
| Low muscle mass (%) | 75 (6.4) | 0 (0.0) | 224 (100.0) | <0.001 |
| Calf circumference (cm) | 36.2 (3.4) | 35.5 (2.9) | 28.99 (2.35) | <0.001 |
| Low walking speed (%) | 138 (11.9) | 385 (57.7) | 193 (86.2) | <0.001 |
| Walking speed (m/s) | 1.14 (0.33) | 0.66 (0.45) | 0.33 (0.36) | <0.001 |

**S2 Table. Total number of observed transitions in participants categorized by sarcopenia over a 12-year follow-up**

|  | **From** | | | |
| --- | --- | --- | --- | --- |
|  | **No**  **sarcopenia** | **Probable**  **sarcopenia** | **Sarcopenia** |  |
| **To** |  |  |  |  |
| **No sarcopenia** | 2360 | 199 | 22 |  |
| **Probable sarcopenia** | 606 | 1019 | 67 |  |
| **Sarcopenia** | 129 | 228 | 242 |  |
| **Death** | 306 | 501 | 411 |  |
| **Loss to follow-up** | 422 | 225 | 58 |  |

*Notes*. Individuals could have experienced more than one transition.

**S3 Table. Mean permanence time in each sarcopenia state**

|  | **Mean permanence time in each state**  **(95% Confidence Interval)** |
| --- | --- |
| **Initial status** |  |
| **No sarcopenia** | 9.69 (9.17-10.24) |
| **Probable sarcopenia** | 4.22 (3.96-4.50) |
| **Sarcopenia** | 2.55 (2.34-2.77) |

**S4 Table. Factors associated with transitions from no sarcopenia, probable sarcopenia, and sarcopenia (univariate analysis)**

|  | **Univariate Hazard Ratios (95% Confidence Intervals) of Transition** | | | | | | | | | | | | |
| --- | --- | --- | --- | --- | --- | --- | --- | --- | --- | --- | --- | --- | --- |
|  | **From no sarcopenia to** | | | | **From probable sarcopenia to** | | | | **From sarcopenia to** | | | | |
|  | **Probable**  **sarcopenia** | **Sarcopenia** | **Death** | **Loss to**  **follow-up** | **No**  **sarcopenia** | **Sarcopenia** | **Death** | **Loss to**  **follow-up** | **No**  **sarcopenia** | **Probable**  **sarcopenia** | **Death** | **Loss to**  **follow-up** |  |
| Age (years) | **1.09**  **(1.08-1.10)** | **1.14**  **(1.12-1.16)** | **1.08**  **(1.07-1.09)** | **1.02**  **(1.01-10.3)** | **0.96**  **(0.94-0.97)** | **1.06**  **(1.05-1.08)** | **1.09**  **(1.08-1.11)** | 1.00  (0.99-1.02) | **0.95**  **(0.91-0.99)** | **0.96**  **(0.93-0.98)** | **1.07**  **(1.05-1.09)** | 0.99  (0.96-1.03) |  |
| Sex (M vs F) | **0.62**  **(0.52-0.74)** | 1.03  (0.73-1.47) | **1.5**  **(1.2-1.87)** | 0.94  (0.77-1.14) | 0.86  (0.61-1.21) | 1.07  (0.79-1.45) | **1.37**  **(1.13-1.66)** | 0.87  (0.63-1.2) | 0.76  (0.28-2.04) | 0.78  (0.44-1.37) | 1.11  (0.9-1.37) | 0.95  (0.54-1.69) |  |
| Education  (high vs low) | **0.77**  **(0.60-0.98)** | **0.42**  **(0.27-0.64)** | **0.59**  **(0.43-0.8)** | **0.55**  **(0.43-0.72)** | 1.15  (0.79-1.68) | 0.67  (0.5-0.91) | **0.59**  **(0.48-0.72)** | 0.97  (0.69-1.36) | 2.17  (0.66-7.16) | 1.22  (0.68-2.16) | 0.83  (0.67-1.02) | 1.34  (0.71-2.53) |  |
| Smoking habits  (ref: never) |  |  |  |  |  |  |  |  |  |  |  |  |  |
| Former | **0.67**  **(0.57-0.80)** | 0.92  (0.62-1.36) | 0.93  (0.72-1.21) | 0.94  (0.76-1.16) | 0.8  (0.58-1.09) | **0.68**  **(0.50-0.91)** | 1.01  (0.83-1.22) | 0.96  (0.72-1.28) | 0.5  (0.16-1.51) | **0.48**  **(0.26-0.89)** | 1.08  (0.87-1.35) | 0.96  (0.53-1.73) |  |
| Current | **0.57**  **(0.44-0.73)** | 1.41  (0.89-2.22) | **1.53**  **(1.14-2.05)** | 1.21  (0.93-1.57) | 1.16  (0.73-1.82) | 0.71  (0.43-1.17) | 1.2  (0.90-1.62) | 1.15  (0.73-1.79) | 1.4  (0.46-4.24) | 0.41  (0.15-1.14) | 0.94  (0.67-1.31) | 1.42  (0.67-2.97) |  |
| Alcohol  (ref: no/occ) |  |  |  |  |  |  |  |  |  |  |  |  |  |
| Light to  moderate | **0.69**  **(0.58-0.82)** | **0.62**  **(0.43-0.9)** | **0.5**  **(0.39-0.64)** | **0.63**  **(0.51-0.78)** | 1.82  (1.34-2.48) | 0.78  (0.6-1.02) | **0.63**  **(0.52-0.76)** | **0.64**  **(0.49-0.85)** | 1.07  (0.45-2.53) | **1.76**  **(1.06-2.92)** | **0.68**  **(0.55-0.84)** | 0.94  (0.55-1.61) |  |
| Heavy | **0.38**  **(0.27-0.53)** | **0.4**  **(0.2-0.79)** | 0.74  (0.52-1.04) | **0.66**  **(0.48-0.91)** | 0.86  (0.37-2.00) | 0.63  (0.31-1.29) | 0.85  (0.57-1.28) | 0.88  (0.49-1.6) | 0.99  (0.13-7.67) | 1.74  (0.61-4.99) | 0.67  (0.39-1.14) | 0.71  (0.17-2.99) |  |
| Physical level  (active vs  inactive) | 0.81  (0.65-1.01) | 0.65  (0.40-1.06) | **0.43**  **(0.34-0.55)** | 0.84  (0.65-1.08) | **2.51**  **(1.65-3.81)** | 0.79  (0.55-1.14) | **0.51**  **(0.42-0.63)** | 0.87  (0.65-1.18) | 1.30  (0.47-3.58) | 1.77  (0.90-3.50) | **0.51**  **(0.39-0.68)** | 1.03  (0.50-2.12) |  |
| MMSE | **0.89**  **(0.87-0.92)** | **0.84**  **(0.81-0.87)** | **0.85**  **(0.83-0.87)** | **0.90**  **(0.87-0.94)** | **1.26**  **(1.16-1.37)** | **0.95**  **(0.93-0.97)** | **0.92**  **(0.91-0.93)** | 1.01  (0.98-1.04) | **1.22**  **(1.04-1.43)** | **1.04**  **(1.00-1.09)** | **0.95**  **(0.94-0.96)** | **1.05**  **(1.00-1.11)** |  |
| BMI (kg/m^2^) | **1.03**  **(1.01-1.05)** | **0.77**  **(0.73-0.81)** | 0.98  (0.95-1.01) | 1.02  (0.99-1.04) | 1.00  (0.96-1.03) | **0.87**  **(0.84-0.91)** | **0.96**  **(0.93-0.98)** | 0.97  (0.94-1.01) | 0.98  (0.86-1.12) | **1.09**  **(1.01-1.17)** | 0.98  (0.95-1.01) | 1.06  (0.98-1.15) |  |
| N. chronic  diseases | **1.16**  **(1.14-1.19)** | **1.21**  **(1.16-1.26)** | **1.18**  **(1.15-1.22)** | 1.01  (0.98-1.05) | **0.88**  **(0.84-0.93)** | **1.05**  **(1.02-1.09)** | **1.12**  **(1.1-1.14)** | 1.01  (0.97-1.04) | **0.81**  **(0.7-0.93)** | 0.98  (0.92-1.04) | **1.07**  **(1.05-1.1)** | 0.98  (0.92-1.05) |  |

*Abbreviations:* Mini-Mental State Examination; M, males; F, females. *Notes*. High educational level includes high school degree or above. Physical level, Mini-Mental State Examination, and the number of chronic diseases are included in the model as time-varying variables. Participants stable in no sarcopenia, probable sarcopenia and sarcopenia stages are the reference categories. Bold values indicate statistically significant estimates (p-value <0.05)

**S5 Table. Factors associated with transitions from no sarcopenia, probable sarcopenia, and sarcopenia to loss to follow-up**

|  | **Multivariable HR (95%CI) of Transition to Loss to Follow-up** | | | |
| --- | --- | --- | --- | --- |
|  | **From No Sarcopenia** | **From Probable**  **Sarcopenia** | **From Sarcopenia** |  |
| Age (years) | **1.02 (1.00-1.03)** | 1.00 (0.99-1.02) | 1.00 (0.95-1.06) |  |
| Sex (male vs female) | 0.90 (0.72-1.14) | 0.86 (0.58-1.28) | 1.12 (0.49-2.56) |  |
| Education (high vs low) | **0.72 (0.55-0.96)** | 0.93 (0.64-1.37) | 1.29 (0.48-3.43) |  |
| Smoking habits (ref: never) |  |  |  |  |
| Former | 1.04 (0.83-1.30) | 1.14 (0.83-1.58) | 0.97 (0.41-2.32) |  |
| Current | **1.32 (1.00-1.75)** | 1.48 (0.91-2.41) | 1.6 (0.59-4.33) |  |
| Alcohol (ref: no/occas.) |  |  |  |  |
| Light to moderate | **0.70 (0.56-0.89)** | **0.61 (0.45-0.83)** | 1.24 (0.58-2.69) |  |
| Heavy | 0.80 (0.55-1.17) | 0.78 (0.36-1.69) | 0.96 (0.18-5.08) |  |
| Physical level  (active vs inactive) | 0.94 (0.73-1.21) | 0.94 (0.68-1.29) | 0.84 (0.40-1.80) |  |
| MMSE | **0.92 (0.88-0.95)** | 1.00 (0.95-1.06) | 1.09 (0.95-1.26) |  |
| BMI (kg/m^2^) | 1.02 (0.99-1.05) | 0.97 (0.93-1.01) | 1.04 (0.92-1.18) |  |
| N. chronic diseases | 0.98 (0.94-1.02) | 1.00 (0.95-1.05) | 0.97 (0.87-1.08) |  |

*Abbreviations:* BMI, Body Mass Index; HR, hazard ratio; 95%CI, 95% confidence intervals; MMSE, Mini-Mental State Examination; occas, occasional. *Notes*. High educational level includes high school degree or above. Physical level, Mini-Mental State Examination, and the number of chronic diseases are included in the model as time-varying variables. Participants stable in no sarcopenia, probable sarcopenia and sarcopenia stages are the reference categories. Bold values indicate statistically significant estimates (p-value <0.05)

**S6 Table. Factors associated with transitions from no sarcopenia, probable sarcopenia, and sarcopenia in community-dwelling individuals (n = 3,104)**

|  | **Hazard Ratios (95% Confidence Intervals) of Transition** | | | | | | | | | | | |
| --- | --- | --- | --- | --- | --- | --- | --- | --- | --- | --- | --- | --- |
|  | **From no sarcopenia to** | | | | **From probable sarcopenia to** | | | | **From sarcopenia to** | | | |
|  | **Probable**  **sarcopenia** | **Sarcopenia** | **Death** | **Loss to**  **follow-up** | **No**  **sarcopenia** | **Sarcopenia** | **Death** | **Loss to**  **follow-up** | **No**  **sarcopenia** | **Probable**  **sarcopenia** | **Death** | **Loss to**  **follow-up** |
| Age (years) | **1.08**  **(1.07-1.10)** | **1.11**  **(1.07-1.14)** | **1.07**  **(1.05-1.08)** | **1.01**  **(1.00-1.03)** | **0.98**  **(0.96-1.00)** | **1.04**  **(1.01-1.06)** | **1.07**  **(1.05-1.08)** | 1.00  (0.99-1.02) | 0.99  (0.93-1.07) | 0.96  (0.93-1.00) | **1.04**  **(1.01-1.06)** | 1.00  (0.95-1.06) |
| Sex (M vs F) | **0.73**  **(0.60-0.90)** | **1.83**  **(1.16-2.89)** | **1.83**  **(1.43-2.35)** | 0.91  (0.72-1.15) | 0.93  (0.62-1.41) | **2.00**  **(1.32-3.03)** | **1.52**  **(1.18-1.95)** | 0.87  (0.58-1.29) | 0.78  (0.23-2.68) | 0.49  (0.20-1.22) | **2.04**  **(1.47-2.82)** | 1.13  (0.49-2.59) |
| Education  (high vs low) | 1.30  (0.98-1.72) | 1.08  (0.60-1.95) | 1.15  (0.83-1.59) | **0.72**  **(0.54-0.95)** | 0.88  (0.56-1.38) | 1.06  (0.65-1.72) | 0.8  (0.63-1.03) | 0.95  (0.65-1.39) | 1.21  (0.33-4.41) | 1.43  (0.59-3.50) | 0.87  (0.63-1.22) | 1.28  (0.48-3.4) |
| Smoking habits  (ref: never) |  |  |  |  |  |  |  |  |  |  |  |  |
| Former | 0.86  (0.71-1.04) | 1.32  (0.81-2.15) | 1.12  (0.86-1.46) | 1.04  (0.83-1.30) | 0.85  (0.60-1.20) | **0.65**  **(0.43-0.98)** | 1.07  (0.85-1.34) | 1.14  (0.83-1.58) | 0.54  (0.14-2.06) | 0.57  (0.25-1.28) | 1.03  (0.74-1.44) | 0.96  (0.40-2.29) |
| Current | 0.88  (0.66-1.17) | **2.12**  **(1.21-3.73)** | **2.01**  **(1.47-2.75)** | **1.33**  **(1.00-1.76)** | 0.96  (0.56-1.62) | 0.77  (0.38-1.57) | **1.70**  **(1.17-2.45)** | 1.49  (0.91-2.41) | 1.14  (0.28-4.54) | 0.50  (0.14-1.70) | 1.13  (0.71-1.80) | 1.57  (0.58-4.25) |
| Alcohol  (ref: no/occ) |  |  |  |  |  |  |  |  |  |  |  |  |
| Light to  moderate | 1.07  (0.87-1.31) | 0.92  (0.57-1.50) | **0.74**  **(0.56-0.96)** | **0.71**  **(0.56-0.89)** | 1.43  (0.99-2.08) | 1.04  (0.71-1.54) | 0.83  (0.67-1.04) | **0.60**  **(0.44-0.82)** | 0.81  (0.28-2.36) | 1.72  (0.87-3.41) | 0.77  (0.56-1.05) | 1.25  (0.58-2.70) |
| Heavy | 0.89  (0.59-1.34) | 0.85  (0.35-2.05) | 0.81  (0.54-1.21) | 0.8  (0.55-1.17) | 0.76  (0.28-2.08) | 0.73  (0.27-1.97) | 1.05  (0.67-1.67) | 0.77  (0.35-1.66) | 0.93  (0.08-10.2) | 1.73  (0.31-9.77) | 0.59  (0.28-1.23) | 0.96  (0.18-5.08) |
| Physical level  (active vs  inactive) | 0.97  (0.78-1.22) | 0.72  (0.43-1.19) | **0.53**  **(0.41-0.68)** | 0.93  (0.72-1.20) | **1.84**  **(1.19-2.84)** | 0.82  (0.56-1.20) | **0.76**  **(0.62-0.94)** | 0.94  (0.68-1.28) | 0.86  (0.29-2.52) | 1.98  (0.91-4.33) | 0.75  (0.55-1.02) | 0.85  (0.40-1.80) |
| MMSE | 1.03  (0.97-1.08) | 0.96  (0.87-1.06) | **0.91**  **(0.87-0.95)** | **0.91**  **(0.88-0.95)** | **1.17**  **(1.05-1.30)** | 1.03  (0.96-1.10) | **0.92**  **(0.90-0.94)** | 1.00  (0.95-1.06) | 1.16  (0.91-1.48) | 1.00  (0.90-1.11) | **0.95**  **(0.92-0.98)** | 1.08  (0.94-1.25) |
| BMI (kg/m^2^) | 1.05  (1.02-1.07) | **0.75**  **(0.70-0.80)** | 0.98  (0.95-1.01) | 1.02  (0.99-1.04) | 1.00  (0.96-1.04) | **0.86**  **(0.81-0.91)** | **0.98**  **(0.95-1.00)** | 0.97  (0.93-1.01) | 1.06  (0.90-1.26) | 1.09  (0.98-1.22) | **0.94**  **(0.89-0.99)** | 1.04  (0.92-1.18) |
| N. chronic  diseases | 1.02  (0.99-1.06) | **1.07**  **(1.00-1.14)** | **1.07**  **(1.03-1.11)** | 0.98  (0.94-1.02) | **0.95**  **(0.89-1.00)** | 1.04  (0.98-1.09) | **1.05**  **(1.02-1.08)** | 1.00  (0.95-1.05) | 0.89  (0.76-1.05) | 1.03  (0.94-1.12) | **1.08**  **(1.04-1.12)** | 0.97  (0.87-1.08) |

*Abbreviations:* HR, hazard ratio; 95%CI, 95% confidence intervals; MMSE, Mini-Mental State Examination; M, males; F, females. *Notes*. High educational level includes high school degree or above. Physical level, Mini-Mental State Examination, and the number of chronic diseases are included in the model as time-varying variables. Participants stable in no sarcopenia, probable sarcopenia and sarcopenia stages are the reference categories. Bold values indicate statistically significant estimates (p-value <0.05)

**Table S7. Chronic diseases associated with transitions from no sarcopenia, probable sarcopenia, and sarcopenia**

|  | **Multivariable Hazard Ratio (95% Confidence Interval) of Transition** | | | | | | | | |
| --- | --- | --- | --- | --- | --- | --- | --- | --- | --- |
|  | **From No Sarcopenia to** | | | **From Probable Sarcopenia to** | | | **From Sarcopenia to** | | |
|  | **Probable**  **sarcopenia** | **Sarcopenia** | **Death** | **No**  **sarcopenia** | **Sarcopenia** | **Death** | **No**  **sarcopenia** | **Probable**  **sarcopenia** | **Death** |
| Cardiovascular diseases | 0.91  (0.69-1.22) | 1.53  (0.89-2.64) | **1.64**  **(1.31-2.06)** | 0.46  (0.20-1.07) | 0.89  (0.47-1.69) | **1.47**  **(1.17-1.85)** | 0  (0- >1000) | 0.68  (0.11-4.17) | **1.78**  **(1.24-2.53)** |
| Neuropsychiatric diseases | 1.14  (0.86-1.50) | 1.31  (0.75-2.28) | 1.16  (0.91-1.48) | 1.13  (0.61-2.08) | 1.04  (0.56-1.92) | 1.20  (0.95-1.52) | 155.92  (0- >1000) | 1.64  (0.28-9.68) | 1.23  (0.86-1.76) |
| Gastrointestinal/kidney  diseases | 1.17  (0.91-1.49) | 0.74  (0.43-1.26) | 1.08  (0.86-1.36) | 1.26  (0.70-2.29) | 1.11  (0.60-2.04) | 1.06  (0.81-1.37) | 6.67  (0- >1000) | 0.75  (0.16-3.54) | 0.88  (0.58-1.34) |
| Respiratory  diseases | 1.21  (0.86-1.69) | 0.82  (0.37-1.81) | **1.67**  **(1.27-2.21)** | 0.82  (0.36-1.84) | 1.64  (0.82-3.29) | 1.09  (0.81-1.47) | 0.01  (0- >1000) | 3.07  (0.39-23.95) | 1.00  (0.64-1.56) |
| Musculoskeletal diseases | **1.32**  **(1.04-1.67)** | 1.18  (0.70-2.00) | 0.96  (0.76-1.21) | 1.01  (0.58-1.74) | 0.85  (0.48-1.50) | 0.98  (0.79-1.23) | 0.00  (0- >1000) | 1.15  (0.21-6.30) | 1.02  (0.71-1.44) |
| Endocrine/hematologic  diseases | 1.11  (0.86-1.42) | 1.22  (0.72-2.07) | **1.39**  **(1.11-1.73)** | 0.74  (0.40-1.39) | 1.30  (0.73-2.33) | 1.24  (0.99-1.56) | 35.97  (0- >1000) | 1.03  (0.22-4.89) | **1.54**  **(1.08-2.20)** |
| Cancer | **1.85**  **(1.26-2.71)** | 0.53  (0.13-2.20) | **1.95**  **(1.39-2.75)** | **5.60**  **(2.83-11.08)** | 1.72  (0.66-4.43) | 1.19  (0.81-1.76) | 0.04  (0- >1000) | 0.32  (0.01-17.84) | **2.13**  **(1.20-3.79)** |

Model adjusted for: age, sex, education, smoking and drinking habits, physical activity level, and body mass index (at baseline). Participants stable in no sarcopenia, probable sarcopenia and sarcopenia stages are the reference categories. Bold values indicate statistically significant estimates (p-value <0.05)
